# Spontaneous resolution of acute syphilitic posterior placoid chorioretinitis: reappraisal of the literature and pathogenetic insights

**DOI:** 10.3205/oc000153

**Published:** 2020-05-04

**Authors:** Giuseppe Casalino, Stefano Erba, Vasuki Sivagnanavel, Shervin Lari, Antonio Scialdone, Carlos Pavesio

**Affiliations:** 1 Oftalmico Hospital, ASST Fatebenefratelli Sacco, Milano, Italy; 2 Royal Eye Unit, Kingston Hospital NHS Foundation Trust, London, UK; 3 Moorfields Eye Hospital NHS Foundation Trust, London, UK

**Keywords:** acute syphilitic posterior placoid chorioretinitis, natural course, pathogenesis, retinal imaging, syphilis

## Abstract

Acute syphilitic posterior placoid chorioretinitis (ASPPC) is a rare clinical manifestation of ocular syphilis. Spontaneous resolution of this condition has been reported in a few cases.

The aim of this manuscript is to report an additional case and to discuss the possible pathogenesis of this condition by reviewing the current evidence on this subject.

A 45-year-old man presented to us with decreased vision in the right eye secondary to a placoid macular lesion. Fourteen days after presentation, there was a dramatic improvement of the vision, and multimodal retinal imaging showed almost complete spontaneous resolution of the placoid lesion. Syphilis serology turned out positive and a diagnosis of ASPPC was made. The pathogenesis of ASPPC is unclear, and there is contrasting evidence about the role of the cellular immune system. Since this condition may resolve spontaneously before systemic antimicrobial treatment, the presence of a placoid macular lesion should raise a high suspicion of ASPPC in order to make a timely diagnosis and to avoid progression of untreated syphilis.

## Introduction

Syphilis is a sexually transmitted infection caused by the spirochete bacterium *Treponema* *pallidum* [1]. Syphilis is a re-emerging and rising infection in the developed world. In up to one-quarter of patients with syphilis, ocular involvement manifests at any time during the disease course. Ocular syphilis may precede the diagnosis of systemic disease in up to one-half of cases [2]. Ocular syphilis, known as “the great masquerader”, may affect almost every structure of the eye and has a broad spectrum of presentation, including, among others, interstitial keratitis, optic neuropathy and posterior uveitis, the latter commonly represented by chorioretiniti [3], [4].

In 1988, de Souza et al. [5] reported three young patients with “unilateral central chorioretinitis” as manifestation of ocular syphilis. Two years later, Gass et al. [6] reported six additional similar cases. They concluded that this condition was a separate clinical entity, and coined the term “acute syphilitic posterior placoid chorioretinitis” (ASPPC).

ASPPC is defined by the presence of one or more placoid, yellowish, outer retinal lesions, typically involving the posterior pole and the mid-periphery of the retina near the temporal vascular arcade [6]. ASPPC may have a unilateral or bilateral involvement with a presenting visual acuity ranging from 20/20 to no light perception [7]. The advent of multimodal imaging (MMI) of the retina, especially of spectral domain optical coherence tomography (SD-OCT), has made it possible to report pathognomonic features of ASPPC, which include punctate hyperreflectivity in the choroid, disruption and loss of the ellipsoid zone, nodular irregularity of the retinal pigment epithelium, and transient localized subretinal fluid [8], [9].

Since patients with ASPPC usually receive prompt antimicrobial treatment after serologic results, little is known about the natural course of the disease. To the best of our knowledge, only 5 cases of ASPPC with spontaneous improvement have been reported [10], [11], [12], [13]. We report the natural course and the multimodal retinal imaging features of an additional case, and discuss the pathogenetic implications and the importance of early recognition of this rare clinical entity.

## Case presentation

A 45-year-old man with no relevant past medical history presented to the eye casualty service complaining of sudden onset central ‘white ring’ and decreased vision in the right eye (RE) over the past seven days. Best-corrected visual acuity (BCVA) was 6/12 in the right eye and 6/6 in the left eye (LE). Intraocular pressure was 14 mmHg in both eyes. Examination of the RE showed no cells in the right anterior chamber and 1+ vitreous cells; fundus examination revealed a yellow placoid lesion involving the macular area with no signs of vasculitis or retinal necrosis. Examination of the LE was unremarkable.

**Figure 1:**
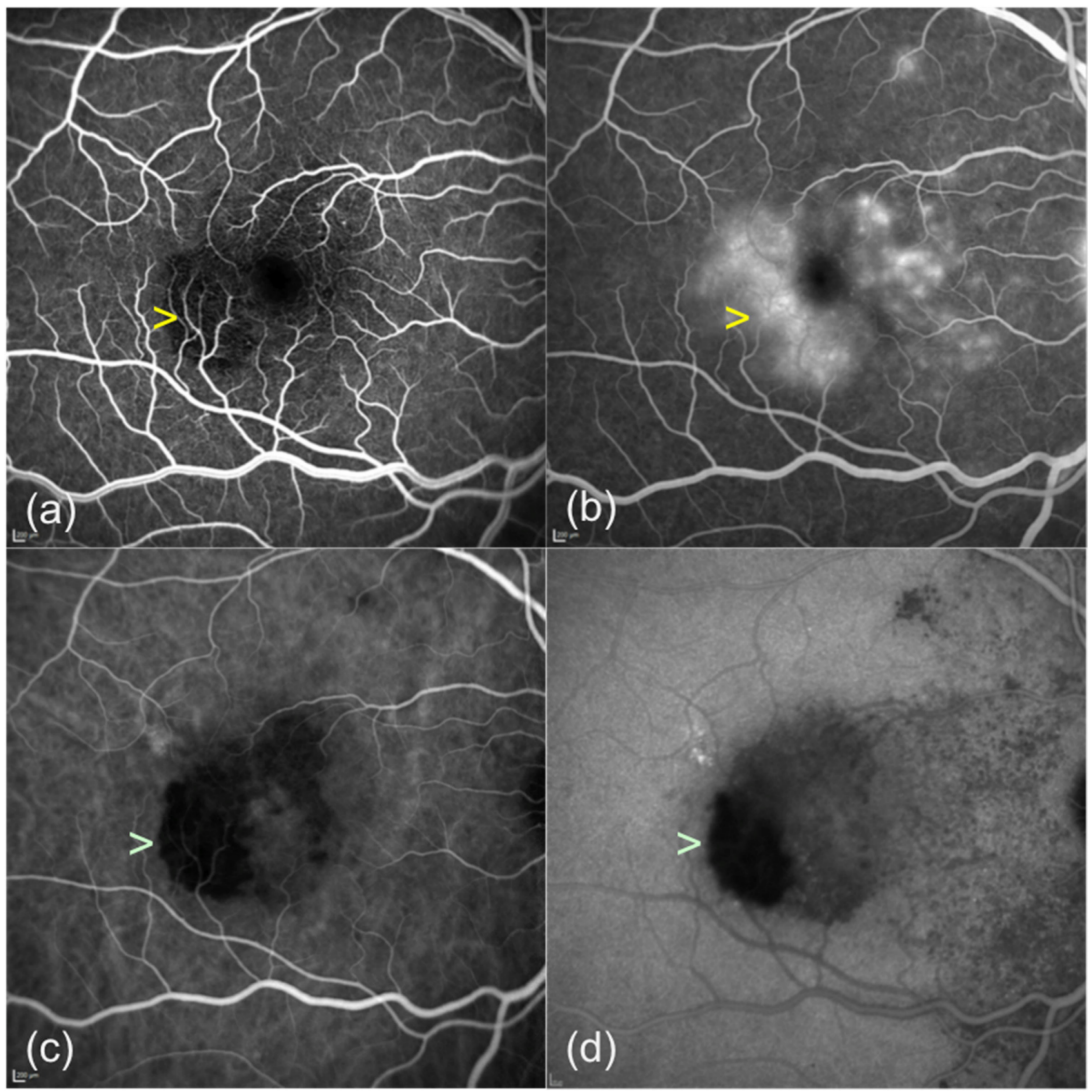
Fluorescein angiography (FA) and indocyanine green angiography (ICGA) of acute syphilitic posterior placoid chorioretinitis in the right eye at presentation. (a) Early frame of FA shows hypofluorescence (yellow arrowhead) of the placoid lesion which appears hyperfluorescent in the late frames (b). (c), (d) ICGA shows hypocianescence of the placoid lesion (green arrowhead) throughout the whole examination.

**Figure 2:**
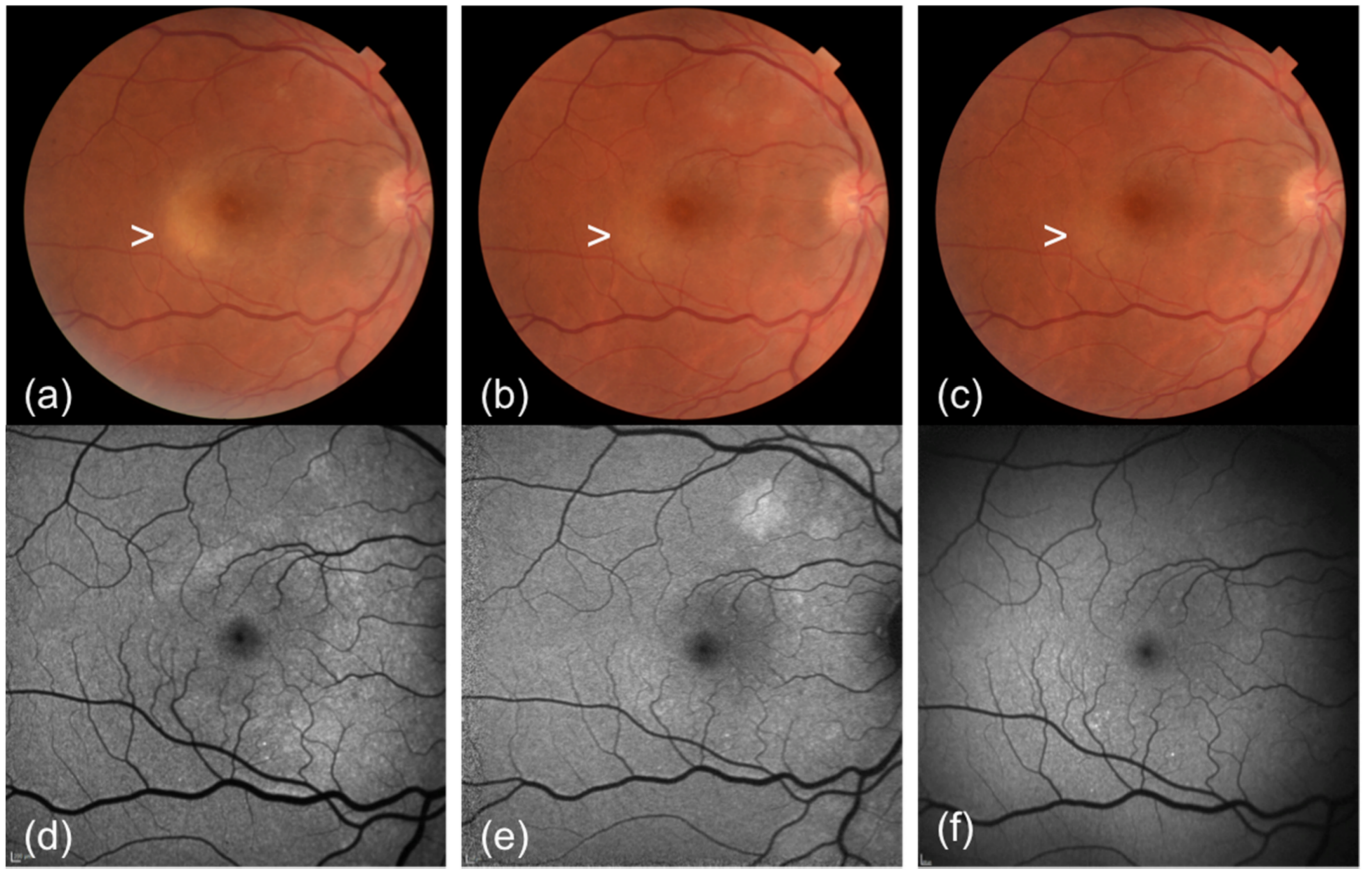
Colour fundus photograph (CFP) and fundus autofluorescence (FAF) changes of acute syphilitic posterior placoid chorioretinitis in the right eye over time. (a) CFP shows a yellow placoid lesion (white arrowhead) at the posterior pole which gradually fades 1 week after presentation (b) and 2 weeks after presentation (c). FAF shows increased AF in correspondence of the placoid lesion at presentation (d) with gradual normalisazion of the AF 1 week after presentation (e) and 2 weeks after presentation (f).

**Figure 3:**
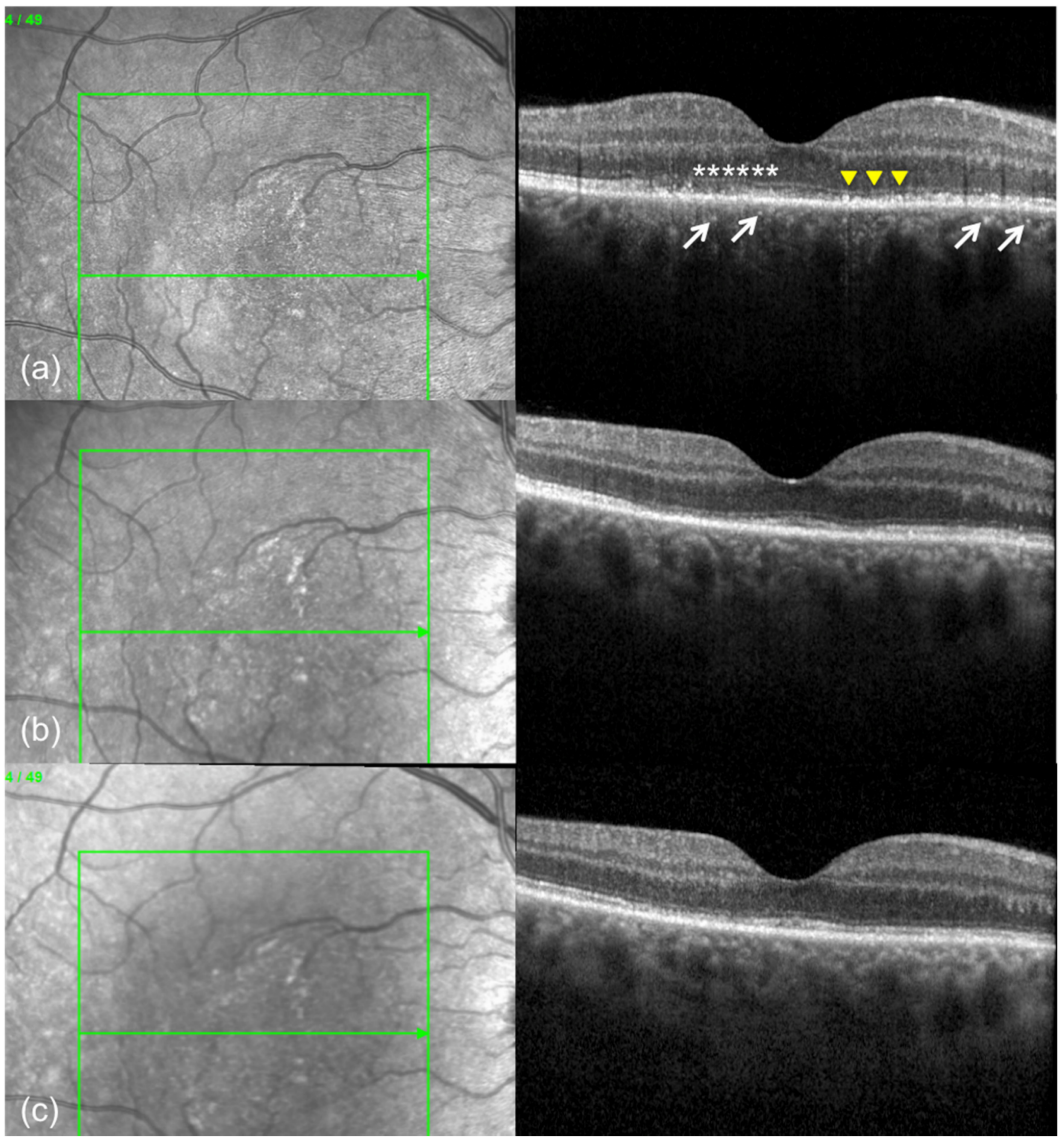
(a) Spectral domain optical coherence tomography (SD-OCT) scan of the right eye at presentation shows disruption of the ellipsoid zone (white asterisks), nodular thickening of the retinal pigment epithelium (yellow arowheads) and punctate hyperreflectivity in the inner choroid (white arrows). SD-OCT scan 1 week after presentation (b) and 2 weeks after presentation (c) show gradual recovery of the ellipsoid zone and retinal pigment epithelium.

MMI of the retina including colour fundus photograph, fundus autofluorescence, SD-OCT, fluorescein angiography and indocyanine green angiography are presented in Figure 1 (Fig. 1), Figure 2 (Fig. 2), and Figure 3 (Fig. 3).

The medical history was carefully reviewed; the patient admitted to be addicted to poppers and cocaine, and reported promiscuous homosexual activity over the last months. He denied intravenous drug use and any systemic symptoms such as headache, skin rash, nausea, weight loss, cough, or night sweats. A complete laboratory work-up was ordered, including TB QuantiFERON-TB testing, syphilis serology and human immunodeficiency virus (HIV) antibodies. Seven days after presentation, the patient reported spontaneous improvement in the vision of the RE, and BCVA improved to 6/9 in the RE and was stable in the LE. Full blood count, liver function test, kidney function, angiotensin-converting enzyme level were within normal range, HIV antibodies were negative. However, results for QuantiFERON-TB testing and syphilis had not been available yet. MMI revealed spontaneous improvement of the placoid lesion (Figure 2 (Fig. 2), Figure 3 (Fig. 3)).

Two weeks after presentation, BCVA further improved to 6/6 in the RE and MMI showed signs of early resolution of the placoid lesion. Laboratory results returned negative for QuantiFERON-TB testing, and positive for venereal disease research laboratory test and fluorescent treponemal antibody testing. Therefore, a definite diagnosis of ASPPC was made, and the patient was promptly referred to the Infectious Disease Department for systemic treatment with penicillin.

## Discussion

ASPPC is a rare clinical manifestation of ocular syphilis. Although the pathophysiology of ASPPC is not completely understood, timing and characteristics of SD-OCT findings may be the reflection of the sequence of disease events [9]. It has been suggested that circulating *T*. *pallidum* organisms may enter the choroidal circulation, giving the choroidal hyperreflective pinpoint lesions seen on SD-OCT; subsequent access to the outer retina may give a variable amount of subretinal fluid and impaired photoreceptor function expressed by disruption of EZ seen on SD-OCT [9].

However, the role played by the cellular immune system in the pathogenesis of this condition remains controversial. While it was initially suggested that ASPPC is secondary to immunocompromised status such as in HIV-positive patients [5], [6], [14], it was later described in both immunocompetent and immunocompromised individuals [7], [9], [15].

Of note, no differences have been found in terms of clinical characteristics and long-term visual outcome in HIV-positive versus HIV-negative patients with ASPPC [7].

To the best of our knowledge, spontaneous resolution of ASPPC before initiation of systemic antimicrobial treatment has been reported in 5 cases [10], [11], [12], [13]. The first cases were described in 2015 by Ji et al. [10], who reported two HIV-negative patients with ASPPC which spontaneously improved 10 days (for the first case) and 3 weeks (for the second case) after presentation. In the same year, Aranda et al. [11] reported an HIV-positive patient on anti-retroviral therapy for 4 years and CD4+ T-cell count of 204 cells/µL presenting with ASPPPC; spontaneous resolution of ASPPC was observed 10 days after presentation [11]. One year later, Franco et al. [12] reported an additional case of ASPPC in an HIV-negative patient with complete spontaneous recovery and no signs of reactivation until the patient was started on antimicrobial treatment, 45 days after presentation.

The spontaneous improvement observed in our case is in line with these previous reports [10], [11], [12] and may suggest that ASPPC is the result of the host’s cellular immune response which may be able to locally control the spirochete infection. Some authors have speculated that the immune privilege of the eye may contribute to the spontaneous resolution of ASPPC.

Alternatively, spontaneous resolution of initial ASPPC can be explained as the disease entering prolonged latency. Indeed it is known that syphilis is characterized by episodes of active disease that are interrupted by periods of latency because of the host’s cellular immune response [1], [12]. Therefore the placoid lesions may disappear in the same way the mucocutaneous syphilitic lesions disappear without treatment during the latent stage of the disease. The latter hypothesis has been supported by Baek et al. [13] who reported an HIV-negative patient presenting with ASPPC which spontaneously improved 7 days after presentation, but unlike the aforementioned cases [10], [11], [12] did not receive systemic antimicrobial treatment and returned 9 months later with progression to posterior uveitis [13].

Of note, in 2014 Armstrong et al. [16] had reported spontaneous evolution of unilateral ASPPC to panuveitis in a patient with HIV co-infection. In their case, panuveitis developed 6 weeks after the initial diagnosis of ASPPC, without spontaneous resolution of the lesion.

These latter cases may suggest that ASPPC could be an early manifestation of posterior uveitis, and in absence of an adequate host’s immune response, such as in patients with untreated HIV co-infection, progression without spontaneous improvement may be observed.

In addition, there is evidence that immunosuppression may be a major stimulator of ASPPC. Zamani et al. [17] reported a case of undiagnosed syphilitic chorioretinitis which evolved to multifocal placoid lesions following immunosuppression induced by corticosteroid therapy. Furthermore, Erol et al. [18] and Song et al. [19] published cases of ASPPC following local intravitreal triamcinolone injections. However, in contrast to these aforementioned cases [17], [18], [19], Ormaechea et al. [20] reported a patient with ASPPC who was initially misdiagnosed as having non-infectious uveitis and received corticosteroid as well as methotrexate for 7 months and demonstrated no worsening of the disease.

## Conclusions

There is contrasting evidence about the role of the immune system in the pathogenesis of ASPPC which may be the manifestation of different pathogenetic pathways that ultimately lead to an inflammatory response driven by the presence of the spirochete. Since this condition may resolve spontaneously before antimicrobial treatment, the presence of a placoid macular lesion should raise a high suspicion of ASPPC, as the ophthalmologist may be the first to diagnose syphilis in the patient. Indeed, timely diagnosis and antimicrobial treatment are essential to preventing the progression of syphilis which may include irreversible visual damage [21].

## Notes

### Competing interests

The authors declare that they have no competing interests.

### Informed consent

We obtained written informed consent from the patient for publishing the information and images.

## References

[1] Hook EW (2017). Syphilis. Lancet.

[2] Klein A, Fischer N, Goldstein M, Shulman S, Habot-Wilner Z (2019). The great imitator on the rise: ocular and optic nerve manifestations in patients with newly diagnosed syphilis. Acta Ophthalmol.

[3] Moradi A, Salek S, Daniel E, Gangaputra S, Ostheimer TA, Burkholder BM, Leung TG, Butler NJ, Dunn JP, Thorne JE (2015). Clinical features and incidence rates of ocular complications in patients with ocular syphilis. Am J Ophthalmol.

[4] Amaratunge BC, Camuglia JE, Hall AJ (2010). Syphilitic uveitis: a review of clinical manifestations and treatment outcomes of syphilitic uveitis in human immunodeficiency virus-positive and negative patients. Clin Experiment Ophthalmol.

[5] de Souza EC, Jalkh AE, Trempe CL, Cunha S, Schepens CL (1988). Unusual central chorioretinitis as the first manifestation of early secondary syphilis. Am J Ophthalmol.

[6] Gass JD, Braunstein RA, Chenoweth RG (1990). Acute syphilitic posterior placoid chorioretinitis. Ophthalmology.

[7] Eandi CM, Neri P, Adelman RA, Yannuzzi LA, Cunningham ET, International Syphilis Study Group (2012). Acute syphilitic posterior placoid chorioretinitis: report of a case series and comprehensive review of the literature. Retina.

[8] Brito P, Penas S, Carneiro A, Palmares J, Reis FF (2011). Spectral-domain optical coherence tomography features of acute syphilitic posterior placoid chorioretinitis: the role of autoimmune response in pathogenesis. Case Rep Ophthalmol.

[9] Pichi F, Ciardella AP, Cunningham ET, Morara M, Veronese C, Jumper JM, Albini TA, Sarraf D, McCannel C, Voleti V, Choudhry N, Bertelli E, Giuliari GP, Souied E, Amer R, Regine F, Ricci F, Neri P, Nucci P (2014). Spectral domain optical coherence tomography findings in patients with acute syphilitic posterior placoid chorioretinopathy. Retina.

[10] Ji YS, Yang JM, Park SW (2015). Early resolved acute syphilitic posterior placoid chorioretinitis. Optom Vis Sci.

[11] Aranda S, Amer R (2015). Sequential spontaneous resolution of acute syphilitic posterior placoid chorioretinitis. Eur J Ophthalmol.

[12] Franco M, Nogueira V (2016). Severe acute syphilitic posterior placoid chorioretinitis with complete spontaneous resolution: The natural course. GMS Ophthalmol Cases.

[13] Baek J, Kim KS, Lee WK (2016). Natural course of untreated acute syphilitic posterior placoid chorioretinitis. Clin Experiment Ophthalmol.

[14] McLeish WM, Pulido JS, Holland S, Culbertson WW, Winward K (1990). The ocular manifestations of syphilis in the human immunodeficiency virus type 1-infected host. Ophthalmology.

[15] Joseph A, Rogers S, Browning A, Hall N, Barber C, Lotery A, Foley E, Amoaku WM (2007). Syphilitic acute posterior placoid chorioretinitis in nonimmuno-compromised patients. Eye (Lond).

[16] Armstrong BK, Pitcher J, Shah R, Brady C, Perlmutter D, Garg SJ (2014). The evolution of untreated acute syphilitic posterior placoid chorioretinitis captured by multimodal retinal imaging. Ophthalmic Surg Lasers Imaging Retina.

[17] Zamani M, Garfinkel RA (2003). Corticosteroid-induced modulation of acute syphilitic posterior placoid chorioretinitis. Am J Ophthalmol.

[18] Erol N, Topbas S (2006). Acute syphilitic posterior placoid chorioretinitis after an intravitreal triamcinolone acetonide injection. Acta Ophthalmol Scand.

[19] Song JH, Hong YT, Kwon OW (2008). Acute syphilitic posterior placoid chorioretinitis following intravitreal triamcinolone acetonide injection. Graefes Arch Clin Exp Ophthalmol.

[20] Ormaechea MS, Hassan M, Nguyen QD, Schlaen A (2019). Acute syphilitic posterior placoid chorioretinopathy: An infectious or autoimmune disease?. Am J Ophthalmol Case Rep.

[21] Park JH, Joe SG, Yoon YH (2013). Delayed diagnosis of ocular syphilis that manifested as retinal vasculitis and acute posterior multifocal placoid epitheliopathy. Indian J Ophthalmol.

